# The association between mechanical temporal summation, state anxiety at baseline, and persistent low back pain: a 12-month prospective cohort study

**DOI:** 10.1186/s12891-023-07046-w

**Published:** 2023-12-08

**Authors:** Nicole Dietrich, Hannu Luomajoki, Sabina Hotz-Boendermaker

**Affiliations:** 1 School of Health Professions, Zurich University of Applied Sciences (ZHAW), Institute of Physiotherapy,, Katharina-Sulzer-Platz 9, Postfach, CH-8401 Winterthur, Switzerland; 2 Hirslanden Klinik Linde, Blumenrain 105, CH-2501 Biel, Switzerland; 3 Pain in Motion Research Group, http://www.paininmotion.be

**Keywords:** Wind-up ratio, State anxiety, Pain, Low back pain, Mechanical temporal summation, Linear mixed models

## Abstract

**Background:**

Persons with acute low back pain (LBP) have a good prognosis for regaining function, while pain often persists. Neurobiological and psychosocial factors are recognized to amplify pain responses, as reported for central sensitization. This study investigated the combination of mechanical temporal summation (TS) chosen to characterize central sensitization and state anxiety representing a psychological factor and their association with persistent pain.

**Methods:**

A longitudinal prospective cohort study including 176 participants aged between 18 and 65 with acute LBP was performed. The following independent variables were analyzed at baseline: The mechanical TS at the lower back, of whom the Wind-up ratio (WUR) was calculated, and the state anxiety level measured with the State and Trait Anxiety Inventory (STAI-S). The outcome pain intensity was assessed at baseline and 2,3,6 and 12 months after the onset of acute LBP with the Numeric Rating Scale 0–10 (NRS). Linear mixed models (LMM) were used to analyze the association of the independent variables with pain intensity over time.

**Results:**

The mean baseline WUR was 1.3 (SD 0.6) for the right and 1.5 (SD 1.0) for the left side. STAI-S revealed a mean score of 43.1 (SD 5.2). Pain intensity was, on average, 5.4 points (SD 1.6) on the NRS and decreased over one year to a mean of 2.2 (SD 2.4). After one year, 56% of the participants still experienced pain. The LMM revealed a considerable variation, as seen in large confidence intervals. Therefore, associations of the independent variables (WUR and STAI-S) with the course of the outcome pain intensity over one year were not established.

**Conclusion:**

This investigation did not reveal an association of mechanical TS and state anxiety at baseline with pain intensity during the one-year measurement period. Pain persistence, mediated by central sensitization, is a complex mechanism that single mechanical TS and state anxiety cannot capture.

## Background

Low back pain (LBP) is the most common musculoskeletal disorder, with a prevalence of 7.3% of the world's population [1]. Although it is a leading cause of disability, a person with acute LBP has a good prognosis for regaining function. However, several long-term studies have demonstrated that about two-thirds of people experience mild pain after one year [2]. According to the current guidelines, persistent pain lasts longer than three months. Unfortunately, persistent pain is associated with a significant social and economic burden [3, 4].

Various factors contribute to the progression from acute nociceptive pain arising from tissue damage to the persistence of symptoms in persons with chronic LBP. In general, peripheral and central sensitization are recognized to enhance pain responses on the neurophysiological level. While peripheral sensitization in the acute state is perceived as a physiological adaptation for tissue healing, it should recede once the structure's healing is completed and pain decreases [5]. Conversely, central sensitization is a neurophysiological mechanism within the central nervous system, resulting in pain hypersensitivity [6, 7]. These amplified functions of neurons and neural networks that modify temporal, spatial, and threshold changes in pain responses may account for pain persistence. With such adaptations in the properties of the neurons in the central nervous system, pain is no longer exclusively coupled to noxious peripheral stimuli but to somatosensory inputs that usually evoke pain-free sensory phenomena [6, 7]. The clinical manifestation of central sensitization is described as increased pain sensitivity, such as hyperalgesia or allodynia, expansion of the original pain area, or enhanced temporal summation (TS) [7, 8]. TS describes a spinal mechanism where repetitive noxious stimuli with uniform intensity increase pain perception [9]. Slow TS can be seen as a behavioral correlate to "wind up," a response related to the hyperexcitability of dorsal horn neurons [10]. However, TS has a broader function, as it can trigger additional processes such as habituation or descending inhibition [11]. Therefore, generating the TS phenomenon to measure the maladaptive perception of pain could be seen as a marker of central sensitization, particularly in chronic pain [12, 13].

A meta-analysis of persons with LBP confirmed these findings. The comparison between healthy individuals and persons with LBP revealed enhanced pain responses to mechanical TS in the lumbar region [14]. Besides, in ongoing LBP, higher pain intensity in mechanical TS predicted a worse outcome concerning physical functioning and movement-evoked pain in a one-week follow-up [15]. However, the extent of these effects could depend on the methods used for TS [16]. As these studies were performed with persons with chronic LBP, it remains to be investigated whether endogenous pain modulation can already be observed in the acute stage.

Early detection of such processes could have a predictive value concerning the development of persistent pain. Furthermore, brain imaging in healthy participants has revealed that TS resulted in brain activity patterns involved in somatosensory processing and various areas responsible for cognition, affect, and pain modulation [17]. A review involving persons with chronic LBP extended these findings and reported on altered central nociceptive processing when analyzing brain functions [16]. In line, Klyne et al. (2019) assessed sensory profiles (i.e., pain threshold and conditioned pain modulation) together with psychological data to predict recovery of LBP after six months. They concluded that sensory measures alone were not predictive of pain persistence and suggested that testing should be combined with psychological assessments to identify persons with high sensitivity and negative psychological states [18]. Negative emotions and adverse beliefs have been previously reported as mediators for central sensitization, resulting in increased pain perception [9, 12]. The prevalence of mental disorders, such as depression or anxiety, increases in persons with chronic pain, including LBP [19, 20]. Although not fully understood, it is acknowledged that psychological distress and anxiety predict persistent pain in persons with LBP [19]. According to Spielberger's anxiety theory, anxiety can be divided into state and trait anxiety. Trait anxiety is the relatively stable interindividual difference in the tendency to evaluate situations as threatening, and, in such situations, state anxiety increases as a reaction. State anxiety thus captures a subjective, consciously perceived feeling of fear and tension accompanied by the agitation of the autonomous nervous system representing distress [21]. Hallegraeff et al. (2020) reported state anxiety to predict persistent LBP for a 12-week measurement time [22]. However, since LBP often persists beyond one year, the state anxiety predictive value for this period is of great interest.

Thus, after tissue damage, neurobiological and biopsychosocial factors can alter its sensitivities, which may lead to augmented pain transmission as reported for central sensitization [6, 16]. This knowledge should instigate clinicians to think beyond muscles and joints [23]. Thus, clinicians need a cost-effective and practical approach to measuring pain sensitivity in clinical assessments [23]. Therefore, this study investigated whether mechanical TS applied with an everyday object in the acute phase (< 4 weeks) was associated with increased LBP intensity over one year. In addition, the state's anxiety dynamic reaction as a proxy for distress should also be assessed as a potential confounding variable. The findings could enhance our understanding of central sensitization in acute LBP. Eventually, the results define a straightforward approach for clinicians to determine a feature of central sensitization in acute LBP. Ultimately, the findings could strengthen a more direct assessment of hyperactivity in pain processing in primary care and secondary prevention of persistent LBP.

## Methods

### Study overview

This study was embedded in a large prospective cohort study investigating clinical, somatic, and psychosocial factors of LBP. Subject's data were collected within the first month of the onset of acute LBP (baseline; T1), at two months (T2), at three months (T3), at six months (T4), and after twelve months (T5) by online surveys and physical assessments. This study reports on the measures of the TS applied by mechanical stimuli, the state anxiety level at baseline, and their association with the outcome pain intensity over one year (T1-T5). The study protocol was in accordance with the Declaration of Helsinki, and approval was obtained from the Ethics Committee of the Canton of Zurich (BASEC-No. 2016–02,096). All procedures followed relevant guidelines, such as the Strobe checklist and other regulations.

### Participants

The cohort consisted of 176 participants. Inclusion criteria were aged between 18 and 65 years and acute LBP for less than four weeks. Participants needed to be pain-free for at least three months in case of recurrent pain episodes. Participants were required to have internet access and be able to read and understand the German language. Exclusion criteria were psychiatric disorder, the use of psychiatric medication, birth less than twelve months ago, pregnancy, and specific pathologies of the back, such as tumors, infections, and unstable anomalies. Drop-outs were defined as missing data in two subsequent measurements, an unreliable answer of more than one-week delay, or withdrawal from the study.

### Recruitment

The participants were recruited from two outpatient clinics, private physiotherapy practices, and a university campus. They were recruited via the university campus homepage, intranet, flyers, advertisements, or email. An informed consent form was signed, and the inclusion criteria were assessed before the first examination.

### Measurements

#### Clinical examination

Experienced physiotherapists carried out clinical examinations. To ensure the standardization of the assessments, the examinators were trained for two hours for all physical measurements.

For the examination of the mechanical TS, a wooden toothpick was used [24], although there are more validated assessment tools available. The German Research Network has standardized the measurement of mechanical TS on Neuropathic Pain (DFNS) using a pinprick stimulator applying a force of 512 Nm [25]. In clinical investigations, this measuring device was repeatedly employed [15]. An alternative presents the Von Frey Hairs (Aesthesio No. 6.45) that apply mechanical stimuli with 180Nm [26]. In addition, the Neuropen was evaluated as a cost-effective alternative for pinprick stimulators [27]. Still, the wooden toothpick was the most economical and time-saving fashion to assess mechanical TS. Investigations on toothpick TS have been conducted. Due to unevenly distributed patient groups, its validity could not be confirmed [28]. Although the wooden toothpick lacks quality criteria, its application as a measurement device for mechanical TS has been recommended. The guidelines for Pain-Orientated Sensory Testing (POST) advise using a toothpick in clinical practice and examinations [29]. Similarly, toothpicks are recommended for Quantitative Sensory Testing in physiotherapeutic settings [24].

For the WUR calculation, the pain intensity value after 10 stimulations was divided by the pain intensity after the first stimulation. However, in many participants, the first stimulation was painless. The value 0 in the denominator of the ratio calculation results in mathematically invalid values. Thus, for the computation of the WUR, we applied a mathematical formula [30]. Researchers have been dealing with this dilemma in different ways. By defining the first stimulation as painful (i.e., NRS score 1), the pain intensity was above zero [31]. A further option was the removal of participants who indicated no pain in the first stimulation from data analysis [20, 32]. In the first case, it is questionable how the standardized strength of the stimulation can be maintained. In the latter case, the omission of participants can lead to bias. Thus, it stands to reason that the definition and implementation of the WUR should be analyzed and standardized in future research.

The stimulation's location was at the level of L4, 5 cm lateral to the processus spinosus within an area of 1 cm^2^. The intensity of the pressure was chosen to make an initial dent visible in the skin without damage to the skin surface. Pain intensity was assessed after one stimulation and ten subsequent stimuli with a one-second interval [24, 28]. The participants indicated the pain intensity on a Numeric Rating Scale (NRS) from 0 to 10, where 0 represents "no pain" and 10 "most intense pain imaginable."

The Wind-up ratio (WUR) was then calculated according to the German Research Network on Neuropathic Pain (DFNS) [25]. The pain rating of the ten stimulations was divided by the first stimulus pain rating. For the calculation of the WUR, mathematically invalid values can occur if the subject indicates 0 on the NRS, i.e., a painless stimulation during the first stimulation. The following mathematical approximation to the WUR formula solved this dilemma, as previously described by Allison and colleagues [30].

$$\frac{pain intensity after 10 stimulations+ 1}{pain intensity after 1 stimulation +1}$$

### Online survey

The participants completed an online survey the same week the clinical examinations were conducted. The survey recorded sociodemographic data and, by using different questionnaires, assessed physical and psychological complaints. Medication for the current LBP episode was collected. Participants could check off general pain medication (nonsteroidal anti-inflammatory drugs, Paracetamol), opioids, antidepressants, muscle relaxation, and others.

#### Pain intensity

The outcome pain intensity was measured using three NRSs for the current pain, the average pain over the past week, and the maximum pain over the past week [33]. The pain intensity was computed as an average of these three values reflecting the overall pain situation [33].

#### State and Trait Anxiety Inventory (STAI-S)

The State Anxiety Scale of the State and Trait Anxiety Inventory (STAI-S) was employed to measure state anxiety. The subscale contains 20 items, each screened on a four-point Likert scale, whereby a sum score between 20 and 80 points can be computed. A higher value indicates a higher state of state anxiety and can be applied as a proxy for distress. The subscale has a good to excellent internal consistency with a Cronbach alpha of 0.88–0.96 but a wide range of retest reliability with 0.03–0.76 [21].

#### LBP associated disability

The Oswestry Disability Index (ODI) quantifies overall functional disability. The German version of the ODI has excellent test–retest reliability (*r* = 0.96) and strong correlations with the Roland Morris Disability Questionnaire (*r* = 0.8) in patients with chronic pain [34]. Each item was presented as a 6-point Likert scale (from 0 to 5, with 5 indicating the most disability). A score was computed from the points given and then divided by the maximum value (50 points). If one question remains unanswered, the maximum value drops to 45 points, and the score can be evaluated normally [34]. The resulting score is then multiplied by 100 to provide a percentage. Higher scores indicate a more severe disability: 0 to 20% mild disability, 20 to 40% moderate disability, 40 to 60% severe disability, 60 to 80% disabling, and 80 to 100% bedridden or functional impairment.

#### Depression Anxiety Stress Scale (DASS)

The 21-item Depression Anxiety Stress Scale (DASS) questionnaire was designed to assess the severity of depression, anxiety, and stress using three subscales [35]. For the present investigation, we applied the depression and anxiety subscale only. Each subscale’s sum score was computed independently and then multiplied by two to account for the short version of the DASS [35]. Depressive symptoms have a cutoff of > 10 sum score on the subscale depression, with high internal consistency (Cronbach’s alpha 0.91) and construct validity (*r* = 0.68) with the Beck Depression Inventory. The cutoff for anxiety is > 6 sum score on the subscale anxiety [36].

### Data analysis

The distribution of sociodemographic and clinical characteristics of the participants at baseline was analyzed using descriptive statistics. As the disability index’ mean of the cohort was below 20%, indicating none to mild disability, the ODI index was not used for further analysis. Similarly, the mean sum scores for the subscales of depression and anxiety were below the cutoff, indicating marginal mental health issues; these subscales were not considered for further analysis. Still, the results section presented the values when describing the cohort.

A cross-sectional analysis was performed at baseline for the two independent variables, WUR and state anxiety. The outcome pain intensity was analyzed separately at all five measurements (T1-T5).

Linear mixed models (LMM) were fitted to estimate the association of the independent variables WUR bilaterally, and time (2,3,6, and 12 months) with pain intensity over time. Since the variable WUR was measured bilaterally, two models were created: The first for analyzing the association of WUR_R and state anxiety with pain intensity over time and the second for the same associations of WUR_L. Interactions of WUR_R resp. WUR_L and months were included to account for the effect of time. The interaction of WUR_R resp. WUR_L and state anxiety was added to account for the effect of distress on temporal summation. WUR_R resp. WUR_L, state anxiety, and time (months) and their interactions, were considered fixed effects. ID as subject identification was included in all models as a random effect to account for interindividual variability. The following equation describes the model with its variables and parameters:

### LMM 1

Pain intensity_ij_ = β0 + β1 x WUR_R + β2 x STAI-S + β3 x months + β4 x WUR_R x STAI-S + β5 x WUR_R x months + β6 x STAI-S x months + ID_j_ + ε_ij_.

### LMM 2

Pain intensity_ij_ = β0 + β1 x WUR_L + β2 x STAI-S + β3 x months + β4 x WUR_L x STAI-S + β5 x WUR_L x months + β6 x STAI-S x months + ID_j_ + ε_ij_.

i = subject i.

j = timepoint j = 1–5.

β0 = Intercept.

ID = subject (random effect).

ε = error.

Results are presented as coefficients with *p* values and a 95% confidence interval (CI). The significance level was set at *p* < 0.05. SPSS 28.0 (Statistical Package for the Social Sciences) was used for the LMM statistical analyses.

## Results

### Subject characteristic

Recruitment was carried out from November 2017 to March 2020. 176 participants were enrolled at baseline, 161 (91%) were available at two months, 148 (84%) at three months, 133 (76%) at six months, and 126 (72%) at 12 months. Except for one patient having back surgery, all losses to follow-up were missing at random (insufficient adherence to the schedule of clinical tests or questionnaire completion and pregnancy). There were no differences between participants and dropouts at baseline. Table 1 displays an overview of the demographic background and the clinical characteristics of the participants. As the collected clinical data for disability, depression and anxiety were without any clinical relevance, they were excluded from further statistical analysis.

**Table 1:** Baseline characteristics of the participants (*N* = 176)

	**Mean (SD) or %**	**Range**
**Demographic characteristics**		
Age (years)	40.0 (12.8)	19 to 65
Sex (% female)	46.4%	
**Clinical Characteristics**		
**Low back pain**		
Pain intensity (NRS, range = 0–10)	5.4 (1.6)	1 to 10
Number of previous episodes of LBP		
Never	21.8%	
1-2x	29.7%	
3-4x	21.8%	
More than 4x	20.6%	
No answer	6.1%	
**Medication for LBP**		
NSAID (% yes)	34.9%	
Opioid (% yes)	1.8%	
**Disability**		
ODI (0–100%)	16.9% (10.6%)	0 to 53%
Disability to work (in days)	1.8 (3.8)	0 to 30
**Temporal summation: Wind-up ratio**		
*WUR left side*	1.5 (1.0)	0.5 to 8
*WUR right side*	1.3 (0.6)	0.4 to 4
**Psychological characteristics**		
State Anxiety (STAI-S 20–80)	43.1 (5.2)	28 to 54
DASS Depression (0–42)	5.2 (6.4)	0 to 34
DASS Anxiety (0–42)	3.5 (4.8)	0 to 24

*NRS* Numeric Rating Scale, *NSAID* Non-steroidal anti-inflammatory drug, *ODI* Oswestry Disability Index, *DASS* Depression Anxietey Stress Scale

### Pain intensity

**Fig. 1:**
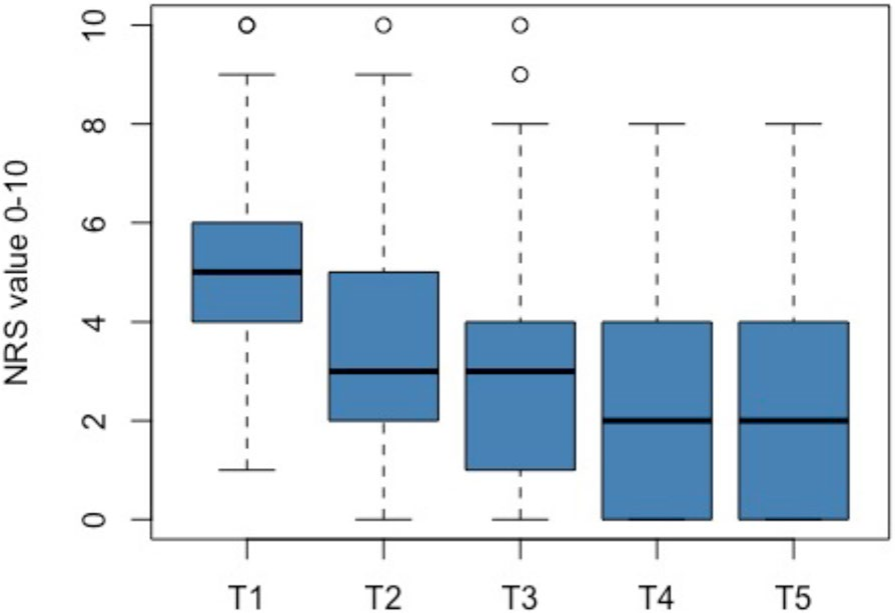
Distribution of pain intensity at all time points. T1 = timepoint 1 (< 4 weeks), T2 = timepoint 2 (2 months), T3 = timepoint 3 (3 months), T4 = timepoint 4 (6 months), T5 = timepoint 5 (12 months)

**Fig. 2:**
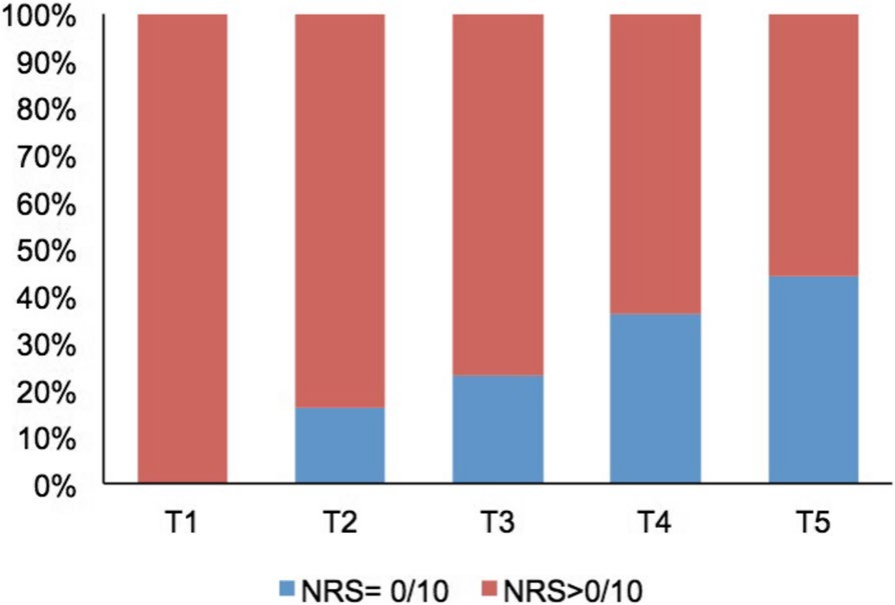
Percentage of participants without pain (NRS = 0/10) vs. participants with pain (NRS > 0/10)**.** T1 = timepoint 1 (< 4 weeks), T2 = timepoint 2 (2 months), T3 = timepoint 3 (3 months), T4 = timepoint 4 (6 months), T5 = timepoint 5 (12 months)

At T1, the participants reported an average pain intensity (measured with NRS) of 5.4 (1.6) points on the NRS. During the following twelve months, the pain intensity decreased continuously to 2.2 at T5. Table 2 shows the course of pain intensity using the mean values and standard deviations, while Fig. 1 shows the distribution on each measurement point with boxplots. Three months after the first onset of symptoms, 77% of the participants still reported pain. This proportion dropped to 64% at T4 and 56% at T5. Thus, half of the participants reported pain one year after the initial manifestation (Fig. 2).

**Table 2:** The linear mixed models for the association of the independent variables (WUR, state anxiety) and their interaction with the outcome pain intensity

	**Coefficient (SE)**	**95% Confidence Interval**		***P*-Value**
**LMM 1**				
Intercept	1.78	-4.97	8.53	0.60
WUR_R, baseline	2.41	-2.25	7.07	0.31
STAI, baseline	0.01	-0.14	0.17	0.86
Months	-0.19	-0.56	0.18	0.31
WUR_R x STAI	-0.04	-0.14	0.07	0.47
WUR_R x months	-0.07	-0.13	0.00	0.04
STAI x months	0.00	0.00	0.01	0.32
**LMM 2**				
Intercept	4.11	-2.51	10.74	0.22
WUR_L, baseline	0.31	-3.82	4.44	0.88
STAI_baseline	-0.02	-0.17	0.13	0.79
Months	-0.21	-0.58	0.17	0.27
WUR_L x STAI	-0.01	-0.10	0.09	0.92
WUR_L x months	-0.03	-0.06	0.01	0.20
STAI x months	0.00	-0.01	0.01	0.41

*WUR* Wind up ratio, *R* Right side, *L* Left side, *STAI_baseline* Score State anxiety subscale

### Association of the independent variables with pain intensity

The results of the LMM are presented in Table 2. In LMM 1, coefficients for the association between WUR_R and the state anxiety with the outcome pain intensity over time were computed, while LMM 2 involved the WUR_L. The estimated coefficients of the LMMs revealed a considerable variation, as seen in large confidence intervals. Except for the interaction of WUR_R x months in LMM1, we cannot exclude the zero effect. The great uncertainty of the data is also evident from the *p*-values.

Based on the LMMs outcomes ( see Table 2), the following two equations can be computed, which describe the association of WUR and state anxiety with pain intensity for the present sample.

#### LMM 1

Pain intensity_ij_ = 1.78 + 2.41 × WUR_R + 0.01 × STAI-S—0.19 × months—0.04 × WUR_R x STAI-S -0.07 × WUR_R x months + 0.00 × STAI-S x months + ID_j_ + ε_ij_.

#### LMM 2

Pain intensity_ij_ = 4.11 + 0.31 × WUR_L—0.02 × STAI-S—0.21 × months—0.01 × WUR_L x STAI-S—0.03 × WUR_L x months + 0.00 × STAI-S x months + ID_j_ + ε_ij_.

i = subject I, j = timepoint j = 1–5, β0 = Intercept, ID = subject (random effect), ε = error.

When considering the values, the following tendencies are noticeable (Table 3): Assuming stable state anxiety: Increased WUR_R increases the pain intensity. However, this effect decreases over time. In contrast, with increasing WUR_L, pain intensity decreases. With stable WUR: An increase in state anxiety score reduces pain intensity. These computations explain the association between the baseline WUR and pain intensity over time, which are opposite concerning the side of measurement. Furthermore, state anxiety has no interaction with WUR at baseline and no association with pain intensity over time.

**Table 3:** The tables report pain intensity at different time points associated with fixed adopted values of WUR and state anxiety

Tables for WUR_R and STAI-S						Tables for WUR_L and STAI-S					
Timepoint 2: 2 months						Timepoint 2: 2 months					
^WUR_R^	0.5	1	1.5	2	2.5	^WUR_L^	0.5	1	1.5	2	2.5
_STAI-S_						_STAI-S_					
30	2.24	2.77	3.31	3.84	4.38	30	3.07	3.04	3.02	2.99	2.97
35	2.19	2.62	3.01	3.49	3.93	35	2.94	2.89	2.84	2.79	2.74
40	2.14	2.47	2.81	3.14	3.48	40	2.82	2.74	2.67	2.59	2.52
45	2.09	2.32	2.56	2.79	3.03	45	2.69	2.59	2.49	2.39	2.29
50	2.04	2.17	2.31	2.44	2.58	50	2.57	2.44	2.32	2.19	2.07
Timepoint 3: 3 months						Timepoint 3: 3 months					
^WUR_R^	0.5	1	1.5	2	2.5	^WUR_L^	0.5	1	1.5	2	2.5
_STAI-S_						_STAI-S_					
30	2.01	2.51	3.01	3.51	4.01	30	2.84	2.80	2.76	2.72	2.68
35	1.96	2.36	2.76	3.16	3.56	35	2.72	2.65	2.59	2.52	2.46
40	1.91	2.21	2.51	2.81	3.11	40	2.59	2.50	2.41	2.32	2.23
45	1.86	2.06	2.26	2.46	2.66	45	2.47	2.35	2.24	2.12	2.01
50	1.81	1.91	2.01	2.11	2.21	50	2.34	2.20	2.06	1.92	1.78
Timepoint 4: 6 months						Timepoint 4: 6 months					
^WUR_R^	0.5	1	1.5	2	2.5	^WUR_L^	0.5	1	1.5	2	2.5
_STAI-S_						_STAI-S_					
30	1.34	1.73	2.13	2.25	2.92	30	2.17	2.08	2.00	1.91	1.83
35	1.29	1.58	1.88	2.17	2.47	35	2.04	1.93	1.82	1.71	1.60
40	1.24	1.43	1.63	1.82	2.02	40	1.92	1.78	1.65	1.51	1.38
45	1.19	1.28	1.38	1.47	1.57	45	1.79	1.63	1.47	1.31	1.15
50	1.14	1.13	1.13	1.12	1.12	50	1.67	1.48	1.30	1.11	0.93
Timepoint 5: 12 months						Timepoint 5: 12 months					
^WUR_R^	0.5	1	1.5	2	2.5	^WUR_L^	0.5	1	1.5	2	2.5
_STAI-S_						_STAI-S_					
30	-0.02	0.17	0.36	0.54	0.73	30	0.82	0.64	0.47	0.29	0.12
35	-0.07	0.02	0.11	0.19	0.28	35	0.69	0.49	0.29	0.09	-0.11
40	-0.12	-0.13	-0.15	-0.16	-0.18	40	0.57	0.34	0.12	-0.11	-0.34
45	-0.17	-0.28	-0.4	-0.51	-0.63	45	0.44	0.19	-0.06	-0.31	-0.56
50	-0.22	-0.43	-0.65	-0.86	-1.08	50	0.32	0.04	-0.24	-0.51	-0.79

## Discussion

The longitudinal cohort study investigated the time-related effects and interactions of mechanical temporal summation, operationalized as wind-up ratio (WUR), and state anxiety on pain intensity in acute LBP. Clinical examination and self-reported outcomes were collected simultaneously. Although pain intensity decreased over time, half of the participants reported pain one year after pain onset. Linear mixed models did not reveal an association between WUR and state anxiety with the course of the pain intensity.

### Measuring mechanical temporal summation

Although the wooden toothpick lacks quality criteria, its application as a measurement device for mechanical TS has been recommended [37]. The toothpick is suggested as a cost-effective everyday option in assessing and treating pain in physiotherapeutic settings instead of not performing sensory testing [24]. The mechanical TS-triggered pain was used to calculate the WUR bilaterally. In healthy persons, Rolke et al. reported reference WUR between 2.7 and 3.2 for the face, hand, and foot area, although with a considerable standard deviation. Ratios were not significantly dependent on the body region [25]. Due to different measurement methods and mathematical approaches, the mean values of WUR at baseline (1.3 WUR_R, 1.5 WUR_L) in the present investigation cannot be compared. Furthermore, no reference values exist for persons with acute or chronic LBP. This shortcoming also means that cutoff values for the definition of central sensitization are lacking [7].

### Associations with pain intensity

Central sensitization has been operationalized using a variety of assessments [17]. By applying mechanical TS alone, we aimed to capture the increased central pain processing as a proxy of central sensitization. By adding the affective factor, state anxiety, we assumed to determine perceived fear and tension as a proxy for the autonomous nervous system's agitation. The combination of the two measures was considered to make a strong statement, as suggested by previous evidence [18, 22]. After calculating mixed models, we did detect associations between baseline WUR bilaterally with pain intensity over the first year of LBP. The estimated coefficients of the LMM were relevant but not statistically significant and showed a large confidence interval. Thus, the results impeded the generalization of the findings. Besides, an interaction between WUR and state anxiety was not established. Therefore, a meaningful prediction on evaluating central sensitization, which characterizes the process from acute to persistent pain, was not confirmed using our models.

However, when we filled the results of LMM 1 into the applied equitation, an assumed relationship between WUR_R at baseline with increased pain intensity over time was seen, given that state anxiety was static. For example, comparing two participants' WUR_R at baseline with 1 point and 2 points, respectively, after two months, pain intensity increases by 2.77 resp 3.84 for the latter, resulting in a 1.07 points higher pain intensity for WUR_R = 2 at baseline. The relation of WUR_L with pain intensity is similar. The inverse effect of increased WUR_L with pain intensity can be attributed to negative estimates in the associated variables. Although these associations with pain intensity declined over the measurement period, they may be clinically relevant on an individual level.

Increased baseline values in state anxiety resulted in a decrease in pain intensity over time, given stable WUR. These findings contradict our equitation and the study by Hallegraeff et al. (2020), which examined a similar objective [22]. State anxiety is characterized by tension, inner restlessness, nervousness, and increased autonomic nervous system activation [21]. Therefore, it is conceivable that a paradoxical effect of low and high state anxiety can disrupt the healing process. On the one hand, when the symptoms are paid too much attention, avoidance can limit appropriate pain responses. On the other hand, when symptoms are paid too little attention, such as persistence, the injured structures can be overloaded [38]. This assumption warrants further investigations to complement the multidimensionality of LBP.

### Strengths and weaknesses

The strengths of this study were the large sample, the long observation period of one year with a total of five measurement points, and the simultaneous collection of clinical, somatic, and psychosocial data. An easy and validated measuring instrument was used to assess state anxiety. By choosing the toothpick as the measuring instrument, the practicability and cost-effectiveness were high for recording a mechanical TS. However, the lack of quality criteria and comparability with data from the literature can be perceived as a weakness. The fact that this study was embedded in a larger project meant that the participants had to spend time on the five measurements, which led to a lack of adherence. This can be seen, among other things, in the high dropout rate of 28 percent. Although the drop-outs were at random, this significant loss of participants might impair the generalization of the findings.

## Conclusion

This investigation did not reveal a statistically significant association of WUR and state anxiety at baseline with pain intensity during the one-year measurement period. We conclude that the persistence of pain, mediated by central sensitization, is a complex mechanism that single mechanical and psychological measurements cannot capture. In future research, it would be mandatory to investigate further the association between a more extended test panel measuring central sensitization and the course of pain intensity, with the primary goal of finding early predictors of LBP persistence as secondary prevention. Besides, increasing knowledge may also improve therapeutic approaches.

## Data Availability

The data sets used and analyzed in the current study are available on request from SHB.
